# Analysis of paediatric long-term ventilation incidents in the community

**DOI:** 10.1136/archdischild-2019-317965

**Published:** 2019-12-17

**Authors:** Rasanat Fatima Nawaz, Bethan Page, Emily Harrop, Charles A Vincent

**Affiliations:** 1 Department of Experimental Psychology, University of Oxford, Oxford, UK; 2 Patient Safety Collaborative, Oxford Academic Health Science Network, Oxford, UK; 3 Helen and Douglas House, Oxford, UK

**Keywords:** comm child health, health services research

## Abstract

**Aim:**

To describe the nature and causes of reported patient safety incidents relating to care in the community for children dependent on long-term ventilation with the further aim of improving safety.

**Methods:**

We undertook an analysis of patient safety incident data relating to long-term ventilation in the community using incident reports from England and Wales’ National Reporting and Learning System occurring between January 2013 and December 2017. Manual screening by two authors identified 220 incidents which met the inclusion criteria. The free text for each report was descriptively analysed to identify the problems in the delivery of care, the contributory factors and the patient outcome.

**Results:**

Common problems in the delivery of care included issues with faulty equipment and the availability of equipment, and concerns around staff competency. There was a clearly stated harm to the child in 89 incidents (40%). Contributory factors included staff shortages, out of hours care, and issues with packaging and instructions for equipment.

**Conclusions:**

This study identifies a range of problems relating to long-term ventilation in the community, some of which raise serious safety concerns. The provision of services to support children on long-term ventilation and their families needs to improve. Priorities include training of staff, maintenance and availability of equipment, support for families and coordination of care.

**What is already known on this topic**

- The number of children on long-term ventilation cared for at home is rapidly increasing.
- There are significant risks in long-term ventilation that need to be carefully managed.
- Little is known about the safety of care for children on long-term ventilation in the community.

**What this study adds**

- This study identifies a range of problems in care and underlying factors experienced by children on long-term ventilation at home.
- Priorities for improvement are training of staff, maintenance and availability of equipment, support for families and improving coordination of care.

## Introduction

Children and adults with complex medical needs are increasingly cared for in the community, rather than in hospital.^1^ Long-term ventilation is a mechanical aid for breathing, used either invasively by tracheostomy or non-invasively via a mask interface for all or part of the 24-hour day.^2^ There are increasing numbers of long-term ventilated children and young people living at home, with a wide variety of underlying conditions.^3^ Many are eventually weaned off ventilation, but some, often with life-limiting conditions, remain dependent on long-term ventilation for part or all of their lives. The number of children on home ventilation in the UK increased from 93 children in 1990 to 844 in 2008,^2^ with recent estimates of 1500 children reported in 2015.^3^ There are many potential benefits of community delivered long-term ventilation for the child and family, such as being able to live at home, participate in family life and attend school. However, there are also significant risks, which need to be safely managed such as equipment problems, and staff and parent competency.

### Support for families on long-term ventilation care

Children on long-term ventilation need an extensive care package to provide long-term medical, nursing and physiotherapy support.^4^ Common procedures carried out by parents and staff include changing tracheostomy tapes, suctioning of the tracheostomy, manual ventilation, ventilator care, infection control, stoma care and emergency planning.^5^ Cases of accidental death have been reported in the literature.^6^

Prior to discharge from hospital, family caregivers are expected to undergo extensive in-hospital training to provide essential knowledge and competencies.^7^ There are some examples of rigorous training programmes in the literature, including the use of simulation training for parents.^5 8^ However, there is little standardisation of training or assessment of knowledge and ongoing competency in practice.^9^ In a survey of parents’ and nurses’ knowledge of unexpected situations with tracheostomies or their ventilators at home, 63% did not know about alarms related to accidental dislodgement of the tracheostomy tube and 52% failed to understand high-pressure alarms and mucous plugging.^10^ Information on quality control of ventilator care in the home has shown that only 56% of hospitals initiating home ventilation assessed whether patients or caregivers cleaned and operated the ventilator equipment correctly after discharge.^11 12^

### Learning from incidents

Analysing incident reports offers a window into the safety of systems, highlighting vulnerabilities and inadequacies, and detecting common problems and rare and serious risks.^13^ The aim of this study is to describe the nature and causes of reported patient safety incidents relating to care in the community of children on long-term ventilation and develop recommendations for improving the safety of care.

## Methods

### Data source

The data for this study comes from the National Reporting and Learning System (NRLS). This is a national repository of patient safety incident reports from across England and Wales.^14^ NHS Trusts, individuals and organisations can voluntarily submit reports to the national repository. They are encouraged to report any ‘patient safety incidents’, defined as ‘any unintended or unexpected incident that could have or did lead to harm for one or more patients receiving NHS-funded healthcare’.^14^ The reports are anonymised and include open text boxes for information about what happened and why it happened, as well as categorical information such as patient demographics, level of harm, location and date of the incident. More information on the NRLS data is available on their website.^14^

### Sample selection

A request for all incidents relating to long-term ventilation was sent to NRLS for incidents occurring between January 2013 and December 2017, for patients under 18 years of age. The following search terms were used to identify the incidents: long term vent*, long term ventilation, home vent*, level 3 vent*, level three vent*, lvl 3 vent*, lvl three vent*, trache, trachy and trachi*, where the asterisk is a ‘wildcard’ representing one or more other characters. A total of n=4036 incidents were received from NRLS.

**Figure 1:**
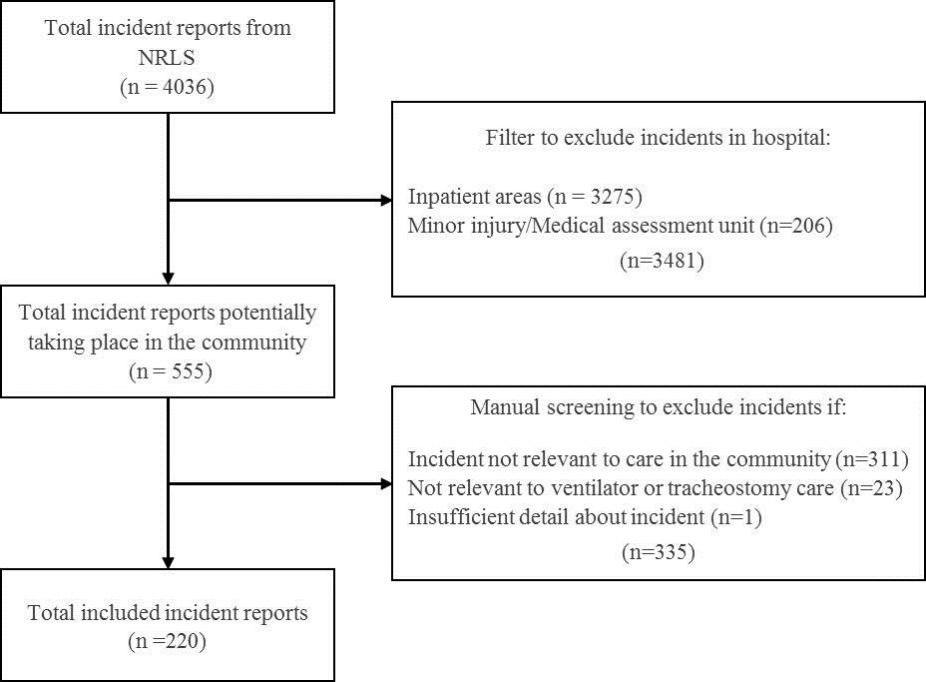
Flow diagram showing the steps taken to identify the final sample of incidents for review.

The incidents were filtered by reported incident location to identify incidents reported in the community. They were then manually screened to remove any further incidents not relevant to ventilator or tracheostomy care, or not occurring in community settings. This produced a final sample of 220 incidents for analysis. Figure 1 shows a flow diagram illustrating the steps taken to identify the sample.

### Analysis

The selected incidents were imported into NVivo, version 12, software for qualitative analysis. The free text boxes for each incident were coded to identify the reported problems in care, any stated contributory factors and any stated patient outcome where evident from the reporter’s narrative. An adapted framework approach was used.^15^ An initial framework was created for problems in care, contributory factors and patient outcomes based on the framework used in a previous study by the same authors, which was developed by adapting existing frameworks for categorising problems in care, contributory factors and patient outcomes primarily in hospital settings.^16^ RFN and BP first independently coded 30 of the incidents identifying the problems in care, any stated contributory factors and any stated patient outcome. The frameworks were then adapted through an iterative process based on the initial coding. The two authors then coded a further 50 incidents independently. Agreement between the authors was good (>95%). Discrepancies in coding were resolved through discussion. The two authors then coded half of the remaining incidents each. Clinical guidance from paediatrician EH was sought when needed. A sample of 10% of the incidents were coded independently by a third author (EH), who has significant clinical expertise in long-term ventilation. Agreement was >90% for the outcomes and care problems and >90% for the contributory factors.

### Ethics

The NRLS data were acquired from the Patient Safety Team and made available through a data-sharing agreement between Oxford Academic Health Science Network (AHSN) and NHS England and NHS Improvement. This service improvement project was a part of the regional Specialist Paediatric Care programme at the Oxford Patient Safety Collaborative at the AHSN.

## Results

### Age breakdown

Incidents were reported across the age span, with 1% reported in babies under 28 days, 22% in children from 1 month to 1 year, 26% in children from 2 to 4 years, 28% in children from 5 to 11 years, 17% in children from 12 to 17 years and 1% in 18 to 25 years (in 5% of incidents the age was unknown).

### Problems identified in the processes of care

At least one problem in care was identified in each incident, with some incidents having two or three problems. Table 1 shows the specific problems identified within each of the categories. The most common problems in the processes of care were issues with faulty equipment and availability of equipment (n=99), factors relating to procedures and treatment (n=91), and concerns around staff availability and competency (n=27). Some of the problems listed under procedures and treatments highlight potential concerns around staff competency (eg, wrong size tube fitted or protocol not followed correctly). There were also 18 instances of problems related to communication and 16 relating to the information, support and training needs of families.

**Table 1:** ​Problems in the process of care

Problems in care	N
**Administration and documentation**	**3**
Notes or documentation errors	2
Notes or documentation not available	1
**Communication**	**18**
Communication failures between staff	9
Communication or handover problems between staff and family	4
Disagreement between staff and family	5
**Discharge**	**4**
Inadequate or no handover from hospital to community teams	2
Required equipment not supplied at discharge	1
Unsafe discharge	1
**Equipment and devices**	**94**
Incorrect equipment ordered or delivered	10
Equipment not available	9
Problem with ventilator circuit	7
No back-up equipment available	6
Equipment delayed or not delivered	4
Emergency tracheostomy bag not with child	4
Faulty or damaged equipment	54
Suction machine	14
Tracheostomy tube	9
Tapes	8
Humidifier	6
Plastic cuff on tracheostomy device	5
Alarm does not sound when it should have	4
Ventilator	4
Circuit	2
Oxygen saturation monitor	2
**Information, training and support needs of families**	**16**
Family carer has not received appropriate training or information	5
Family carer does not follow procedure correctly or goes against advice	4
Concerns relating to family carer’s actions	3
Family carer given inappropriate advice	1
Lack of support for family in the community	1
Child looked after by teenage sibling	1
No training for secondary carers or refresher training for primary carers	1
**Medications**	**8**
Medication or prescription errors, for example, dose errors, missed medication	7
Medication unavailable	1
**Procedures and treatment**	**91**
Tracheostomy tube come out or is dislodged	21
Protocol not followed correctly	15
Wrong size tracheostomy tube fitted	12
Issues with tracheostomy tapes (too loose/tight, wet or skin damage)	10
Water gets in or nearly gets into tracheostomy	9
Child deteriorating	6
Child gets cut when removing tracheostomy tapes	3
Tracheostomy tube is blocked	3
Problems relating to tracheostomy	3
Wrong ventilator settings	1
Child suctioned at wrong length	1
Child desaturates while being cared for by healthcare assistants	1
Child unable to summon help when needing suctioning	1
Spare tracheostomy tube not clean	1
Inappropriate action by nurse following drop in oxygen saturations	1
Too much water inserted into tracheostomy cuff	1
Forgetting to turn the ventilation on	1
Oxygen cylinder is empty	1
**Staffing problems**	**31**
Parents concerned about staff competency	12
Staff asleep while caring for child	9
No staff available	6
Staff do not follow-up problem with family	2
Staff training concerns	2
**Child behaviour**	**8**
Child interfering with equipment or care – query self-harm	6
Child abusive to parents or staff	2
**Parent behaviour**	**3**
Parents aggressive to staff	1
Missed appointment or reviews	1
Parents refuse specialist care support	1
**Other**	4
Transport problems	4

### Outcomes for the child

There was clearly stated harm to the child in 89 (41%) incidents, as identified in the free text descriptions. Table 2 shows the breakdown of outcomes for each incident. Common outcomes resulting in harm to the child included CPR required, emergency tracheostomy change in community setting, and substantial child and parent distress. Some of the incidents in the potential harm category may have resulted in harm to the child which was not stated in the free text. In some of the incidents classified as ‘potential harm’ there was indeed a clear potential for harm, but no actual harm occurred. An example of this would be where the child had the wrong size tracheostomy tube fitted.

**Table 2:** ​Outcomes for child

Outcomes	N
**Clearly stated harm to child**	**89**
Emergency or unplanned tracheostomy change	37
Child severely distressed or in pain	16
Hospital admission or ambulance called	10
Skin damage	8
Distress to parents	6
Child desaturating	4
Other	4
Child may be moribund	2
CPR required	2
**Potential for harm (or harm not stated)**	**131**

### Factors contributing to the incident

There were 50 contributory factors identified in the free text descriptions of the incidents. In most incidents, no specific contributory factors were mentioned by the reporter. Contributory factors fell into six broad categories: family carer factors, equipment factors, organisational factors, patient factors, staff performance factors and environmental factors. Table 3 gives definitions and example quotes for each category. These factors highlight the need for careful assessments and management of risk. Significant risks include challenging behaviour and distress experienced by children, staff shortages and out-of-hours care. Contributory factors relating to equipment highlight potential improvement to design, packaging and instructions.

**Table 3:** ​Types and frequencies of contributory factors with illustrative quotes

Contributory factors	N	Illustrative quotes from incidents
**Family carer factors:** These are features of the family carer or their circumstances that make caring for the child more difficult, or may contribute to problems in care.	4	**Safeguarding concerns:** **“**Report received from the mother of a child who receives continuing care support that the child has been sleeping on an inappropriate piece of equipment. The child is at high risk of developing pressure sores and has been sleeping on a blow-up lilo. He is completely immobile and tracheostomy- and oxygen-dependent (…)There are ongoing child protection concerns which necessitate the requirement for dad’s continued input into the child’s care.” “A long-term ventilated child via a tracheostomy was taken to school without his emergency tracheostomy bag. Sometimes it is a rush to get out in the morning and this may have been a contributing factor.”
**Equipment factors:** These are factors relating to the design of equipment which affect the provision of care.	12	**Factors relating to manufacturing or suppliers** **Issues with packaging:** “Prior to changing trachy tube I noted that the tube I was planning to insert was a different length to the tube I was replacing. The other tube was correct length. The details are very similar on both boxes, which look very similar.” **Crucial information missing from equipment instructions:** “Equipment supplied from manufacturers did not have the cleaning instructions in place, if the filters became wet the device would not work.”
**Organisational factors:** These are features of the way organisations function which affect the provision of care available.	12	**Weekends and out-of-hours:** “Phone call received from LTV (long-term ventilation) patient whose humidifier had failed out-of-hours. As per protocol the parent had called the local hospital where a spare was kept but they were unable to find it.” **Staffing pressures:** “A 24 hours ventilator-dependent tracheostomy child was discharged home following a very prolonged hospital admission. Child under care of children’s long-term ventilation team. Standard of care is that every trache (tracheostomy) long-term vent (ventilation) patient is visited within 24 hours of discharge to follow-up patient and ensure no issues have arisen and troubleshoot any problems. Due to nurse vacancy within the service and only part-time physio within the service the service was unable to offer a home visit until days later.”
**Patient factors**: Features of a patient that make caring for them more difficult and therefore more prone to error.	10	**Communication challenges** **Complex needs:** *“*Community patient who is quadriplegic and is ventilated via tracheostomy was unable to summon help when airway needed clearing while he was at home in bed. He has very limited verbal communication which is worse if airway compromised.” **Child distressed:** “Known risk child will de-cannulate when anxious or displaying behaviours. At 15:15 child was tired and upset and pulled her tracheostomy out.”
**Staff performance factors**: Features of individual staff members that may contribute in some way to problems in care.	10	**Staff panic while changing tracheostomy:** “Child needed to have tracheostomy tapes changed. Staff liaised about who was going to hold the tracheostomy and who was going to change the tapes. Staff member two decided agreed to hold the tube in place while staff member one changed the tapes. While the tapes were being changed, child proceeded to vomit. As staff member one sat child up, staff member two let go of the tracheostomy and staff member one stated that the tracheostomy tube nearly fell out. Staff member one stated that she had shouted at staff member two, with staff member two stating that she had panicked.”
**Environmental factors:** Features of the environment that may contribute to problems in care.	2	**Child is on a plane: “**Child who has long term ventilation was on a flight back from holiday and required ventilation. The portable ventilator failed after 15 min and the child had to be woken up and kept awake so she could breathe unaided.” **Child is in a swimming pool:** “Patient attending pool session at school. Became unwell in the water so removed from the pool by school staff with LTV staff on poolside.”

## Discussion

There are significant risks that need to be managed when caring for children on long-term ventilation in the community. Our analysis of patient safety incident reports found some serious safety concerns. These include issues with faulty and broken equipment, gaps in knowledge and training of staff, and substantial pressure and anxiety experienced by parents. When things do go wrong in the context of long-term ventilation, the consequences for the child and family are potentially very serious. If children with complex care needs are to be cared for safely at home, the provision of services to support these families needs to be improved in several areas. Key recommendations are given in table 4.

**Table 4:** ​Table of recommendations

	Key recommendations
Improving knowledge and training for staff and carers	Promote standardisation of training and spreading of best practice, including competencies, and regular updates. Use simulation training for preparing staff and carers for emergencies such as an unplanned tracheostomy change. Use existing resources^18^ to ensure training for parents is consistently good.
Maintenance and availability of equipment	Ensure correct spare equipment is available and that families know who to call for technical support. Ensure incidents are reported to manufacturers and that design solutions are implemented.
Improving care packages and support for parents	Set national minimum standards in care packages to ensure parents are confident in the care their child is receiving. Make reporting incidents easier for families.
Coordination of support services	Set clear national standards across the patient pathway to improve coordination and communication between services. Ensure commissioners and care providers have high-quality systems in place to train and support those providing high-risk care.

### Improving knowledge and training for staff and carers

A variety of carers look after these children including family members, nurses, paid carers, school and nursery staff, and respite staff. High-risk emergencies can happen at any time, such as blocked or dislodged tracheostomy tubes or ventilator malfunction. Some of the incidents in our study highlight lack of training for staff supporting children on long-term ventilation, including staff panicking in emergency situations. The importance of good quality training and ongoing monitoring of skills for all staff that support these children is paramount for such high-risk care. Simulation training could be more widely used for ensuring staff and other carers can safely manage emergencies.^17^ The charity Well Child have also produced guidelines for training families which provide a useful template for organisations to adopt.^18^

### Maintenance and availability of equipment

Children on long-term ventilation are vulnerable not only to the actions of inadequately trained staff but also to machine failure.^4^ Many incidents in our study were related to faulty or broken equipment which may partly be due to design issues with the equipment, but could also be a result of misuse of equipment by staff or families. In some incidents parents did not have the backup equipment required to perform unplanned tracheostomy changes, leaving the child at risk. There were also instances where information on the packaging did not match the item, as well as similar packaging for different items, leading to incorrect equipment being delivered and used. Problems with the supplies of vital equipment is very stressful for families.^19^ It is important that incident data is brought to the attention of manufacturers so that design solutions can be implemented.

### Improving care packages and support for parents

Many parents report concerns with staff skills or paid carers falling asleep while caring for their child. Other parents reported having to cover multiple night shifts due to staff shortages, while also caring for their child during the day. We need increased standardisation of training for paid carers and enforced standards for care packages highlighting the minimum amount of sleep parents require to safely care for their child. Parents need to be able to hand over responsibility of care without anxiety. It should also be easier for parents to report incidents, with clear follow-up processes, informing parents of any actions taken.

### Coordination of support services

It has been suggested that developments in appropriate community-based services have not kept pace with the medical and technological advances that now allow children with complex needs to be discharged.^20^ The web of services involved needs to be better planned and co-ordinated.^20^ National standards to reduce variability across the patient pathway should be implemented.^21^ It is the responsibility of commissioners and providers of care to have high-quality systems in place to train and support all those providing such high-risk care.

### Limitations

This data represents only a small proportion of the total problems occurring in the community, underestimating the scale of harm.^13 22^ This data tells us about the types of incidents being reported, but it cannot comment accurately on the frequency of safety problems with long-term ventilation. NRLS incidents are predominantly reported through NHS systems meaning third-sector respite care settings, special schools and private agency staff may not be covered directly under the NRLS data repository. Although family carers can directly report incidents themselves, this is very rare in practice. Many of the incidents describing parental concerns are reported through nurses and other healthcare professionals. Ideally parents should also be reporting incidents directly themselves, as they are the primary caregivers.

## Conclusions

This study identifies a range of safety concerns for children on long-term ventilation. Key areas of concern are the training of staff that support these children in the community, the reliability and availability of equipment, the significant stress placed on families and the co-ordination of services. It is important to note that these incidents likely represent the tip of the iceberg. The high-risk nature of the care means that consistently high-quality training for families and for staff is needed. The findings from these incidents emphasise the importance of reporting incidents, including near misses, as there is great value in learning from the data, leading to safer and improved care.
